# Multiple Introductions of the Pestiferous Land Snail *Theba pisana* (Müller, 1774) (Gastropoda: Helicidae) in Southern California

**DOI:** 10.3390/insects12080662

**Published:** 2021-07-21

**Authors:** Jann E. Vendetti, Kimiko Sandig, Armenuhi Sahakyan, Alyana Granados

**Affiliations:** 1 Department of Malacology & Urban Nature Research Center, Natural History Museum of Los Angeles County, 900 Exposition Blvd., Los Angeles, CA 90007, USA; 2 Department of Biology, University of California, Santa Barbara, CA 93106, USA; kimisanciara@gmail.com; 3 Division of Biological Sciences, University of California, San Diego, CA 92093, USA; asahakyan@ucsd.edu; 4 Department of Biology, Williams College, 880 Main St, Williamstown, MA 01267, USA; ajg6@williams.edu

**Keywords:** *Theba pisana*, invasive species, California, provenance, introduced species

## Abstract

**Simple Summary:**

In Southern California, USA, the introduced white Italian land snail, *Theba pisana*, is prolific and locally pestiferous. To better understand its diversity and infer its parent population(s), we collected it from Los Angeles and San Diego counties and generated and analyzed gene sequence data (CO1, 16S, ITS2) that we compared between localities and to *T. pisana* CO1 barcodes from around the world. We also compared the morphology of the jaw, radula, and reproductive systems in *T. pisana* from Los Angeles and San Diego Counties. We found that *T. pisana* living at several sites in Los Angeles County in 2019–2020 had a single origin and were most similar in CO1 DNA sequence, based on available data, to specimens from Malta. *Theba pisana* collected from one site in San Diego County differed from Los Angeles *T. pisana* and were most similar in CO1 barcode sequence to specimens from Morocco. Jaw and mucous gland morphology also differed between Los Angeles and San Diego populations, but it is unclear if these traits are unique to lineages of *T. pisana* or if they change during a snail’s lifetime. We discuss how Los Angeles and San Diego *T. pisana* lineages may have arrived in Southern California and anticipate that the genetic data and morphological observations generated by this study will inform future studies of *T. pisana* where it is native and introduced.

**Abstract:**

The terrestrial land snail *Theba pisana* is circum-Mediterranean in native range and widely introduced and pestiferous in regions around the world. In California, USA, *T. pisana* has been recorded intermittently since 1914, but its source population(s) are unknown, and no morphological or molecular analyses within or between California populations have been published. Therefore, we compared molecular data (CO1, 16S, ITS2) and internal morphology (jaw, radula, reproductive system) in *T. pisana* collected from Los Angeles and San Diego counties in 2019–2020. DNA barcode (CO1 mtDNA) analysis revealed that *T. pisana* from Los Angeles County was most similar to *T. pisana* from the Mediterranean island of Malta, and northern San Diego County-collected specimens were most similar to *T. pisana* from Morocco. Morphology of the jaw and mucous glands also differed between Los Angeles and San Diego populations, but it is unclear if traits are lineage-specific or artifacts of ontogeny. Several pathways of introduction into Southern California are possible for this species, but evidence for intentional vs. accidental introduction of present populations is lacking. Subsequent investigation(s) could use the data generated herein to assess the provenance of *T. pisana* elsewhere in California and/or worldwide and inform analyses of reproductive biology and systematics in this widespread species.

## 1. Introduction

The helicid land snail *Theba pisana* (Müller, 1774) is remarkable for its colonization of regions far beyond its native coastal Mediterranean and Iberian range [1]. As an introduced species, it is found in coastal Argentina [2], several Atlantic islands [1], Northern and Western Europe (e.g., the U.K. and northern France) [3], South Africa [4,5], the U.S. state of California [6], and Australia [7], where it is pestiferous to several agricultural crops [8]. This wide and disjunct distribution has been mostly passive and human-mediated via vehicles and the transport of goods, freight, agriculture, and horticulture [1,9,10,11]. In some cases, its introduction may have been intentional [12,13,14].

In California, USA, *T. pisana* is mostly found in human-altered habitats in Los Angeles and San Diego counties, where it can aestivate in large numbers on structures and plants [15,16]. It was first recorded in California in the community of La Jolla, San Diego County, in 1914 [10,17,18,19], with several sources claiming it was brought from Southern Europe intentionally as an edible “table delicacy” [12,20]. By the early 1920s, the La Jolla population had grown in density and range, extending more than 20.4 hectares (50 acres) [21,22]. It was considered eradicated by the collective efforts of state, county, and federal agencies via vegetation clearing and burning, molluscicide, and hand-picking [17]. However, in 1924, *T. pisana* was found in a cemetery in southeastern San Diego, and in 1936, it was discovered near La Jolla [23]. In the 1980s, it was detected again in the city of San Diego at two separate localities [6,24,25]; it is not known if snails repopulated from survivors of early eradication efforts or if populations resulted from new introductions.

In Los Angeles and Orange Counties, *T. pisana* was recorded between 1934 and 1941 [26,27] in Anaheim, Garden Grove, Huntington Beach, Long Beach, Playa del Rey, San Pedro, and Santa Ana [10,19,28]. In 1935, the Los Angeles community of southeastern San Pedro had at least three dense populations of *T. pisana* that extended more than 40.5 hectares (100 acres), which were supposedly eradicated by burning [29]. In 1966, *T. pisana* was detected in the Los Angeles city of Manhattan Beach [30] and spanned 15 city blocks [31]. It was considered fully eradicated by 1970 after targeted baiting and hand picking [30,32]. 

Since at least 2013 [33], *T. pisana* has been documented in Los Angeles County, mostly in San Pedro [15,34,35], and in many sites in San Diego County from the city of Oceanside to the U.S.-Mexico border district of San Ysidro [16]. Notably, through the 2000s, *T. pisana* was commonly intercepted in cargo (e.g., ceramic tile) and agricultural and horticultural products moving through the ports of Los Angeles, Long Beach, and San Diego [11,36]. The ability of *T. pisana* to aestivate for long periods without water [19], survive high temperatures, and climb and cling to vertical surfaces [37] allows it to persist in Mediterranean climates worldwide and endure long-distance transport. These abilities and its pestiferous habits, e.g., gregarious massing and herbivory, make it a nuisance species and potentially formidable crop pest [19].

Despite the long history and considerable impact of *T. pisana* in Southern California, its source population(s) have not been investigated, nor have any differences between disjunct populations been documented. Several studies have used molecular phylogenetic approaches to examine the provenance and source population(s) of *T. pisana* outside its native range [1,38,39,40], inferring, for example, a French origin for Australian-living *T. pisana* [38] and a Dutch origin for South African-living *T. pisana* [1]. However, none have included California-collected specimens or examined the origins of established populations in Southern California. Therefore, here, we use molecular (mtDNA CO1, 16S rRNA, and ITS2 rDNA) and morphological (reproductive system, radula, and jaw) data to characterize *T. pisana* from populations in Los Angeles and San Diego County, California, to infer their provenance and document their morphological differences.

## 2. Materials and Methods

### 2.1. Specimen Collection

**Figure 1:**
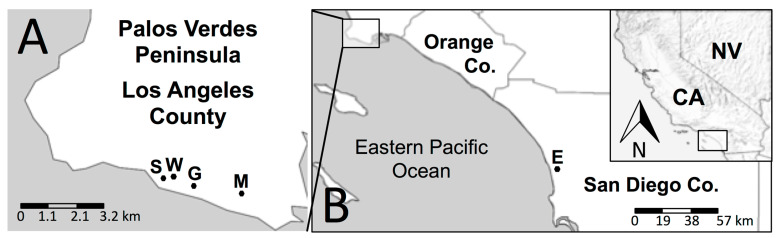
The collection sites for *Theba pisana* (Müller, 1774) in Los Angeles and San Diego Counties, California, USA. (**A**) map of the Palos Verdes Peninsula, Los Angeles Co., S: south entrance of White Point Nature Preserve (WPNP), San Pedro; W: native plant garden, WPNP, San Pedro; G: grassland loop trail of WPNP, San Pedro; M: Marine Mammal Care Center, San Pedro; (**B**) map of southwestern Los Angeles Co. to San Diego Co., California (CA), NV: Nevada, USA; E: Santa Carina trail at San Elijo Lagoon Ecological Reserve, San Diego County.

**Figure 2:**
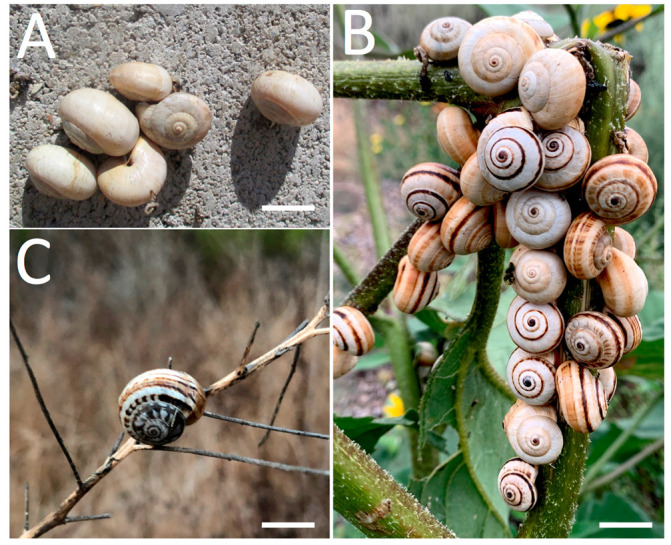
Aestivating *Theba pisana* (Müller, 1774) from sites in Los Angeles and San Diego Counties, California, USA. (**A**) Outside the Marine Mammal Care Center, San Pedro, Los Angeles Co.; (**B**) within the native plant garden of White Point Nature Preserve, San Pedro, Los Angeles Co.; (**C**) along Santa Carina trail, San Elijo Lagoon Ecological Reserve, San Diego Co. Scale bar is 1 cm. Photos A and C by J. Vendetti; photo B by M. Connolly.

Snails fitting the description of *T. pisana* based on shell color and pattern, size, and aggregating (massing) behavior were collected by hand from plants, structures, and in one case, from a Malaise trap intended for insects as part of the BioSCAN community science project [41]. In Los Angeles County, *T. pisana* was collected in 2019 and 2020 from San Pedro at multiple sites (Figure 1 and Figure 2, Table 1). In San Diego County, snails were collected in 2020 from a 5 square meter site along Santa Carina trail within the San Elijo Lagoon Ecological Reserve at the border of the communities of Solano Beach and Encinitas (Figure 1 and Figure 2, Table 1). All collected specimens were killed and preserved 24 h after collection and deposited in the Natural History Museum of Los Angeles County’s (NHMLAC) Malacology Collection, with lot numbers preceded by LACM (Los Angeles County Museum) (Table 1). For clarity and brevity, *T. pisana* specimens will be referred to as “San Pedro” for those collected from San Pedro in Los Angeles County and “San Elijo” for those collected from the San Elijo Lagoon Ecological Reserve in San Diego County.

### 2.2. Specimen Preparation and Morphological Examination

Snails were killed by immersion in carbonated water for 2–4 h, then transferred to and stored in 95% ethanol (EtOH). Dissections under optical microscopy were performed using a Wild Heerbrugg M5A (Switzerland) or Nikon SMZ1000 (Japan) stereomicroscope. Reproductive anatomy was examined by opening each snail’s body near its genital pore and removing its reproductive system. Terminology of reproductive parts follows Gittenberger and Ripken [3], Moran [42], Koene and Schulenburg [43], and Holyoak and Holyoak [44].

Shells and reproductive anatomy were photographed with a Nikon D7200 digital SLR camera using a macro lens. The degree of maturation of reproductive parts was determined by comparing San Pedro specimens collected in June of 2019, June 2020, and October 2020, and San Elijo snails collected in September of 2020. The size of snails’ paired mucous glands (also known as glandulae mucosae) was used to categorize reproductive systems as immature, intermediate, or mature in 5–15 dissected specimens per site (Table 1). Reproductive maturity categories were informed by Moran [42] and were as follows: (1) immature: mucous glands approximately 1 mm long, thin, and rudimentary similar to the rest of the reproductive system; (2) intermediate: mucous glands approximately 5 mm long and 1 mm thick at mid-length; (3) mature: mucous glands 13–16 mm long and 2 mm at mid-length.

Specimens from which radulae and jaws were excised and examined were of typical adult shell size (1.3–1.4 cm) regardless of collection locality. To prepare for scanning electron microscopy (SEM), the buccal mass containing a single jaw and radula was removed from each specimen, immersed in 5M NaOH for 1 to 6 h to dissolve adhering tissue, then cleaned using insect pins and/or fine forceps in several washes of 95% ethanol and water. Clean jaws and radulae were then mounted on metal disks with carbon conductive tape, coated with gold/palladium (60:40) at 0.014 kÅ by an Emitech K550x sputter coater (Kent, United Kingdom), and digitally imaged using a Hitachi S-3000N SEM (Tokyo, Japan) in the SEM laboratory at NHMLAC. Digital image backgrounds were removed in PowerPoint (Microsoft), and image contrast and brightness were adjusted in Preview v. 8.0 in MacOS X. Measurements of jaw width (jw) and jaw ridge length (rl) were made directly from SEM images using its scale bar. Jaw ridge length was calculated as the mean of the two most prominent ridges in each jaw. Jaw width was measured as the length between the middle points of each jaw tip or end.

### 2.3. Generation of Sequence Data

Total DNA was extracted from foot tissue clippings of EtOH-preserved *T. pisana* specimens using the Qiagen DNeasy^®^ Blood and Tissue Kit (Qiagen Corp; Valencia, CA, USA) following the manufacturer’s instructions. Amplification by polymerase chain reaction (PCR) used the following primer pairs: the barcode portion of mtDNA cytochrome c oxidase subunit 1 (CO1): LCO1490 and HCO2198 [45] and jgLCO1490 and jgHCO2198 [46] (*n* = 27, 17 San Pedro and 10 San Elijo), 16S rRNA: 16Sar-L and 16Sbr-H [47] (*n* = 17, only San Pedro), and ITS2 rDNA: LSU-1F and LSU-3R [48] (*n* = 16, 8 San Pedro and 8 San Elijo). Protocols for PCR amplification follow Vendetti et al. [49]. PCR products were purified then sequenced in both directions using PCR primers by Retrogen, Inc. (San Diego, CA, USA). Sequences were visualized as chromatograms and aligned and trimmed of primers in Geneious v. 8.1.6 [50] to a total sequence length of 655 base pairs (bp) for CO1, 402–403 bp for 16S, and 830–835 bp for ITS2. All sequences were submitted to GenBank with the following accession numbers: CO1: MW831635–MW831661, 16S: MW847212–MW847228, and ITS2: MW832220–MW832235. Additional collection details are in Table 1.

### 2.4. Sequence Comparison, Analysis, and Phylogenetic Inference

CO1 barcodes generated for this study were compared to *Theba* taxa within the NCBI GenBank and Barcode of Life (BOLD) databases and were identified therein as *T. pisana*, *T. pisana pisana* (e.g., GenBank acc. nos. HQ864655–HQ864657), *T. p. ampullacea* (e.g., GenBank acc. nos. HM034564–HM034566) and *T. p. cantinensis* (e.g., GenBank acc. no. HM034569). These sequences have been published elsewhere [2,39,40,51] and represent a broad sample of *T. pisana* where native (e.g., Spain, Morocco) and introduced (e.g., South Africa and Australia). Partial CO1 sequences were collapsed into haplotypes using the web interface of FaBox’s DNAcollapser [52]. Genetic distances for CO1 haplotypes were calculated in MEGA7 [53] using the Kimura 2-parameter model (K2P) for nucleotide alignments (using all codon positions).

Mitochondrial CO1 and 16S sequences were concatenated in Geneious v. 6.1.6 and v. 8.1.6 using Geneious Alignment, Consensus Alignment, MUSCLE with default parameters, and the concatenation function. Akaike information criterion (AIC) was used to evaluate the best-fitting model of sequence evolution for CO1, 16S, and ITS2 within jModelTest2 [54] or ModelTest-NG [55] on XSEDE within the CIPRES Science Gateway 3.3 [56]. The outgroup for the concatenated mtDNA CO1 and 16S alignment and analysis was *Theba subdentata*, GenBank acc. nos. MF564172 (CO1) and MF564126 (16S) [57]; for ITS2 it was also *T. subdentata*, GenBank acc. no. KJ458640 [58], in a dataset that also included *T. pisana*, GenBank acc. no. KR705081 [59]. To visualize the relationships among CO1, 16S, and ITS2 sequences, median-joining [60] haplotype networks were created in PopART (Population Analysis with Reticulate Trees) 1.7 [61] using an epsilon value of zero.

Phylogenies were generated in RAxML v. 8.2.12 [62] and MrBayes 3.2.7a [63] within the CIPRES Science Gateway 3.3 [56]. RAxML analyses were run using RAxML-HPC BlackBox with default parameters. Bootstrap values were halted by RAxML automatically after 504–1000 replicates using an MRE-based bootstrapping criterion [64]. MrBayes Bayesian Inference analyses were parameterized to produce a 50% majority consensus tree from two runs of four Markov chains, each of 30 million generations, using the best fitting model of sequence evolution, partitioned with concatenated data, sampling every 1000 trees, and with a discarded burn-in fraction of 25%. For CO1, a second MrBayes analysis was run with the same parameters as described above to account for potential saturation of the third codon position [39]. The resulting phylogenies were visualized using FigTree v. 1.4.3 [65]. Nodes were labeled with maximum likelihood (RAxML) bootstrap support values ≥ 75, followed by Bayesian posterior probabilities ≥ 0.90.

## 3. Results

### 3.1. Shell Morphology

**Figure 3:**
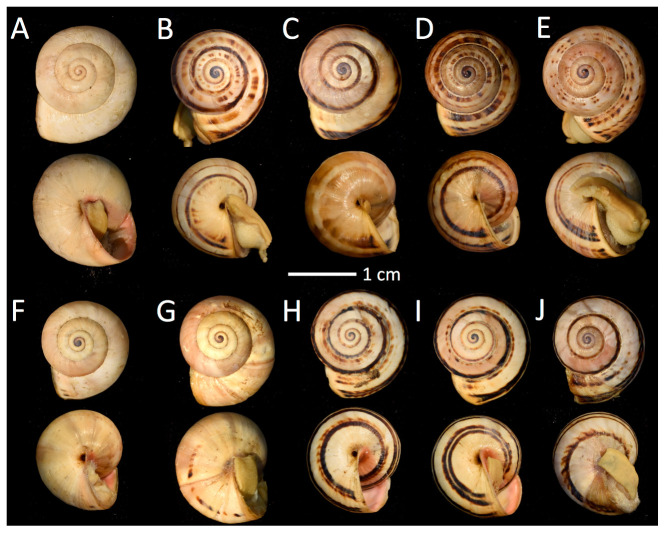
Apical and umbilical view of *Theba pisana* (Müller, 1774) collected in San Pedro, Los Angeles Co. (**A**–**E**) and San Elijo Lagoon Ecological Reserve, San Diego Co. (**F**–**J**), California, USA. (**A**) from outside the Marine Mammal Care Center, LACM 182330; (**B**–**E**) White Point Nature Preserve, LACM 182331; (**F**–**J**) from Santa Carina trail at San Elijo, LACM 182335.

*Theba pisana* shells from San Pedro and San Elijo all were of non-glossy texture, had fine growth lines, an umbilicus partially covered by the body whorl, and lacked a keel or thickened apertural lip. Protoconchs were yellow/white, dark brown, or blue/black. Color and pattern were variable; shells were partially to entirely light yellow to an off-white shade similar to the ground color with a pattern, if present, of light to dark brown stripes, chevrons, and/or mottled or dotted bands (Figure 2 and Figure 3). Characterization of banding (or lack thereof), as well as variation within and between collection sites, was not analyzed quantitatively; however, San Pedro specimens collected near the Marine Mammal Care Center had white to yellow shells with no banding, unlike the mostly patterned and banded shells at all other localities (Figure 3).

### 3.2. Radular Morphology

**Figure 4:**
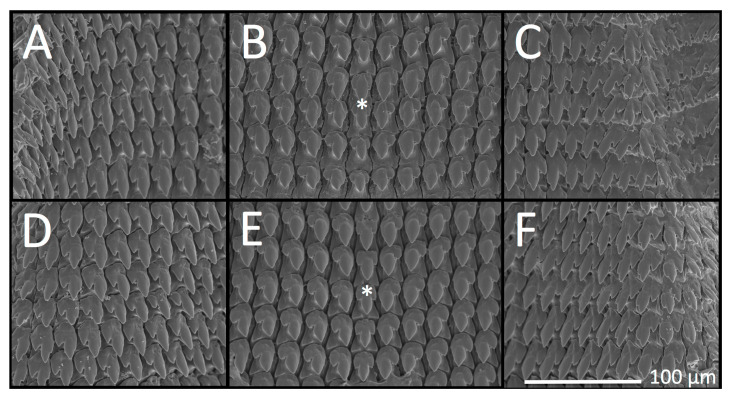
*Theba pisana* (Müller, 1774) radulae as SEM images from specimens collected in San Pedro, Los Angeles Co. (**A**–**C**) and San Elijo Lagoon Ecological Reserve, San Diego Co., California, USA (**D**–**F**). (**A**–**C**) from outside the Marine Mammal Care Center, LACM 182330; (**A**,**C**) lateral and marginal teeth; (**B**) central and lateral teeth; (**D**–**F**) from Santa Carina trail, LACM 182335; (**D**,**F**) lateral and marginal teeth; (**E**) central and lateral teeth. Asterisks indicate a central tooth within the central vertical tooth row.

*Theba pisana* radular morphology examined and figured herein (Figure 4) is consistent with illustrations by Hesse [66] and Barker and Efford [67] and did not differ between San Pedro and San Elijo populations. Tooth size did not differ significantly within or between San Pedro and San Elijo populations: San Pedro central tooth (*n* = 5), length: M = 26.8 μm, SD = 2.05 μm; width: M = 16.40 μm, SD = 0.55 μm; San Elijo central tooth (*n* = 5), length: M = 25.6 μm, SD = 0.55 μm; width: M = 15.60 μm, SD = 0.89 μm. Mature central teeth were symmetrical, tricuspid, and lanceolate with pointed lateral cones and mesocones of a slightly ovate shape that was half the length of the total tooth. The tooth base was an equilateral or isosceles triangle from the tips of the central tooth’s lateral cones to just beyond the tip of the mesocone. Lateral teeth were bicuspid, pointed, and ovate, with a well-developed ectocone half the length of the total tooth. Teeth became lanceolate with a very small endocone that appeared as a bump or tiny point the closer the lateral teeth were to marginal teeth. Marginal teeth were somewhat variable, with a pointed mesocone next to an endocone that could equal it in size, such that the mesocone appeared bicuspid; the ectocone was shorter than the endocone, pointed, and separated from the mesocone by a v-shaped indentation.

### 3.3. Reproductive Anatomy

**Figure 5:**
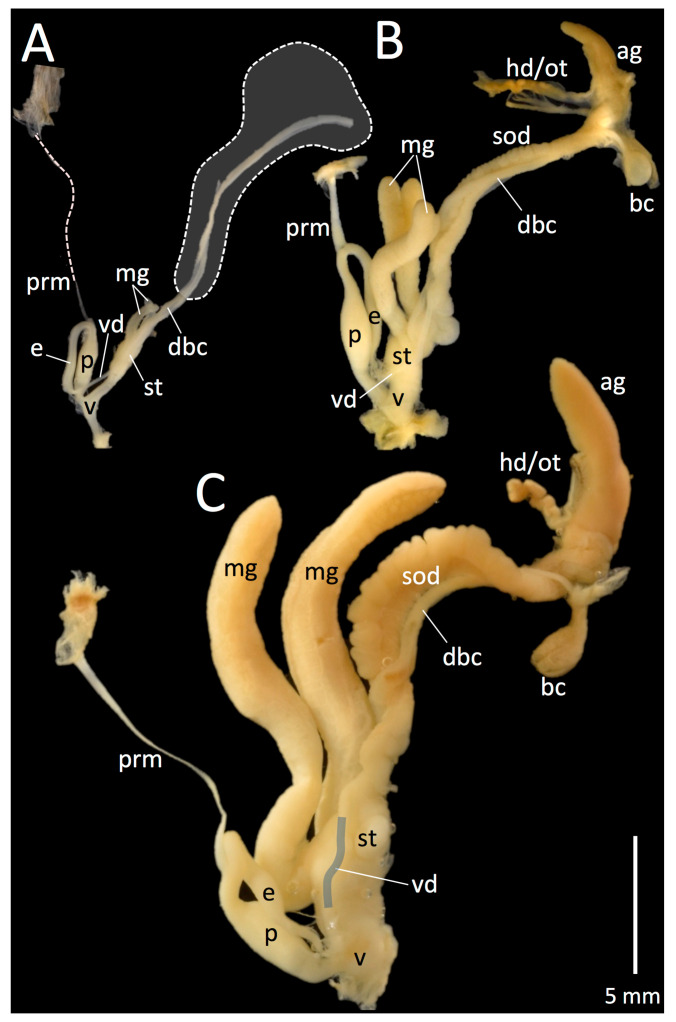
*Theba pisana* (Müller, 1774) reproductive systems at different stages of maturity from San Pedro, Los Angeles Co. (**A**,**C**) and San Elijo Lagoon Ecological Reserve, San Diego Co., California, USA (**B**). (**A**) LACM 182330 (immature), Marine Mammal Care Center, 22 June 2020; (**B**) LACM 182335 (immature?), Santa Carina trail, 12 September 2020; (**C**) LACM 182342 (mature), Grassland Loop Trail, White Point Nature Reserve, 17 October 2020. Abbreviations: ag: albumen gland; bc: bursa copulatrix; dbc: duct of bursa copulatrix; e: epiphallus; hd/ot: hermaphroditic duct/ovotestis; mg: mucous gland; p: phallus; prm: phallus retractor muscle; sod: spermoviduct; st: stylophore (dart sac); vd: vas deferens; v: vagina. In (**A**), the single dashed line indicates part of the phallus retractor muscle; the gray area within the dashed line indicates missing anatomy, including the sod, bc, ag, and hd/ot; in (**C**), the vas deferens is indicated in gray. Anatomical terminology is based on Koene and Schulenburg [43] and Holyoak and Holyoak [44]. Figure layout is based on Moran [42].

Three putative stages of maturation in reproductive anatomy (e.g., immature, intermediate, and mature) were observed in collected *T. pisana* (Table 1, Figure 5). All examined San Pedro specimens collected in June 2019 and June 2020 were reproductively immature (LACM 182329, 182330, 182331, 182358, *n* = 50), September 2020-collected San Elijo *T. pisana* were 20% immature and 80% intermediate (LACM 182335, *n* = 15), and October 2020-collected *T. pisana* from White Point Nature Preserve were 7% intermediate and 93% mature (LACM 182342, *n* = 15). In San Pedro in 2020, the 117 days between collecting events in summer (22 June) and fall (17 October) was sufficient time for most snails’ reproductive systems to mature. However, because specimens were collected only once in San Elijo (in September) and had what we speculate to be mostly intermediate reproductive systems, we do not know their maturation rate or timeline of reproductive maturity. Notably, mucous gland morphology was different between Los Angeles and San Diego sites, with San Pedro specimens having single-lobed mucous glands in October and San Elijo specimens having bi-lobed mucous glands in September.

### 3.4. Jaw Morphology

**Figure 6:**
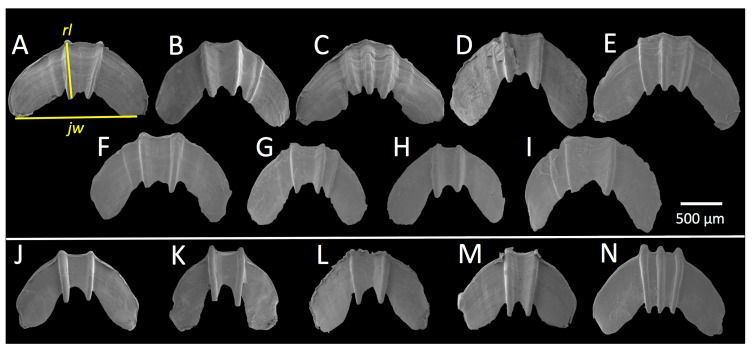
*Theba pisana* (Müller, 1774) jaws as SEM images from specimens collected in San Pedro, Los Angeles Co. (**A**–**I**) and San Elijo Lagoon Ecological Reserve, San Diego Co. (**J**–**N**), California, USA. (**A**–**B**) From the south entrance of White Point Nature Preserve, 22 June 2020, LACM 182329; (C) from outside the Marine Mammal Care Center, 22 June 2020, LACM 182330; (**D**–**I**) from White Point Nature Preserve, 17 October 2020, LACM 182342; and (**J**–**N**) from San Elijo Lagoon Ecological Reserve, 12 September 2020, (**J**) LACM 182335, (**K**–**N**) LACM 182334. Measurements of jaw width (jw) and jaw ridge length (rl) are indicated in (**A**).

Jaws were dark orange to reddish-brown in color, crescent or horseshoe-shaped, and odontognathic with very fine and closely spaced striations and 2–3 medially positioned ridges or ribs of varying prominence and spacing (Figure 6). In all specimens examined, at least two ridges protruded beyond the lower edge of the jaw. Jaw morphology of San Pedro (Figure 6A–I) and San Elijo (Figure 6J–N) specimens appeared to be different; jaw ridges were more prominent and in greater relief in San Elijo specimens than in San Pedro specimens. Ridges in the jaws from five out of five San Elijo specimens were of equal or nearly equal size, and jaws had no minor or less prominent ridges, whereas, in nine out of ten San Pedro specimens, jaws had a less prominent ridge (with specimen H as the exception, Figure 6H). Mean (M) jaw ridge length and mean jaw width differed between San Pedro and San Elijo *T. pisana*, but not significantly: San Pedro jaw width: *n* = 10, M = 1.34 cm, SD = 0.19 cm; jaw ridge length: *n* = 10, M = 0.62 cm, SD = 0.04 cm; San Elijo jaw width: *n* = 5, M = 1.17 cm, SD = 0.12 cm; jaw ridge length: *n* = 5, M = 0.69 cm, SD = 0.09 cm. Jaw ridge prominence, measured as the ratio of jaw ridge length (the mean of the two most prominent ridges in each jaw) to jaw width (the length between the middle points of each end or tip of the jaw’s horseshoe shape), was significantly different between San Pedro and San Elijo *T. pisana* according to a Welch’s *t*-test, t(5.18) = −2.69, *p* < 0.04.

### 3.5. Molecular Sequence Data and Phylogenetic Systematics 

**Figure 7:**
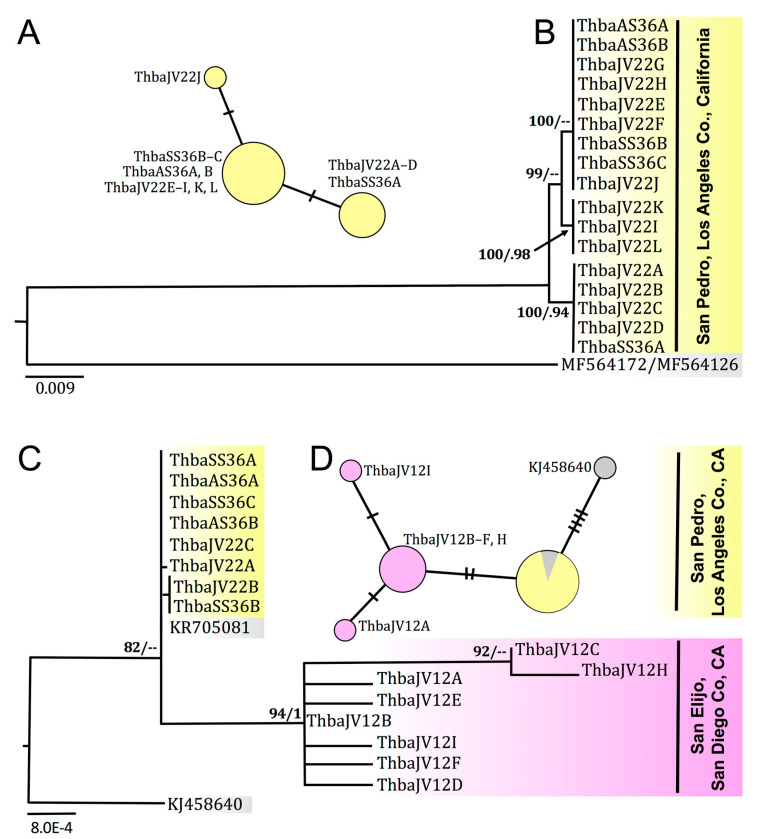
Haplotype networks and RAxML maximum likelihood phylogenies of *Theba pisana* mtDNA CO1 and 16S haplotypes and ITS2 sequences. (**A**) Median-joining 16S rRNA network of 17 *T. pisana* haplotypes from specimens collected in San Pedro, Los Angeles County, California, USA, (**B**) RAxML phylogeny based on concatenated mtDNA CO1 and 16S rRNA sequence data (1059 bp) from specimens collected in San Pedro, Los Angeles County, California (in yellow) with the outgroup *Theba subdentata*, GenBank acc. nos. MF564172 (CO1) and MF564126 (16S), from Souss-Massa, Morocco [57], (**C**) RAxML phylogeny based on partial ITS2 sequence data (836 bp) from specimens collected in San Pedro, Los Angeles County, California (in yellow) and San Elijo Lagoon Ecological Reserve, San Diego County, California (in pink) with *T. pisana*, GenBank acc. no. KR705081, from Tenerife, Canary Islands [59] and the outgroup *Theba subdentata*, GenBank acc. no. KJ458640, from Almería, Spain [58]; (**D**) Median-joining ITS2 network of 16 *T. pisana* sequences from San Pedro, Los Angeles County, California (in yellow) and San Elijo Lagoon Ecological Reserve, San Diego County, California (in pink) with *T. pisana* (KR705081) and *T. subdentata* (KJ458640) as in (**C**). In phylogenies, RAxML bootstrap support values ≥75 are followed by Bayesian posterior probabilities ≥0.90. In haplotype networks, circle size is proportional to the number of haplotypes in the dataset, and each hashmark represents a single nucleotide difference between haplotypes. For GenBank accession numbers, see Table 1.

San Pedro *T. pisana* 16S haplotypes differ by only 1–2 bp (Figure 7A), and CO1 haplotypes differ by 4 bp. In the CO1 and 16S concatenated dataset of San Pedro *T. pisana* (Figure 7B), haplotypes form three clades, the largest of which is reflected in the 16S haplotype network (Figure 7A) and includes specimens from sites within the White Point Nature Preserve but not from the site near the Marine Mammal Care Center (Table 1). Mean intraspecific K2P genetic distance within San Pedro and San Elijo *T. pisana* CO1 haplotypes was 0.4%; between San Pedro and San Elijo populations, it was 13% (Table 2). Between San Pedro and San Elijo *T. pisana* CO1 haplotypes, there were 79 bp differences. Models of sequence evolution for phylogenetic analyses herein were GTR + I + gamma for CO1, K80 for 16S, and TPM1uf for ITS2.

In ITS2 sequence data, phylogenetic analysis resolved Los Angeles and San Diego *T. pisana* as monophyletic, with 82 and 94 bootstrap support, respectively (Figure 7C). ITS2 sequences differed between San Pedro (*n* = 8) and San Elijo (*n* = 8) *T. pisana* specimens by 2 bp (Figure 7D).

**Figure 8:**
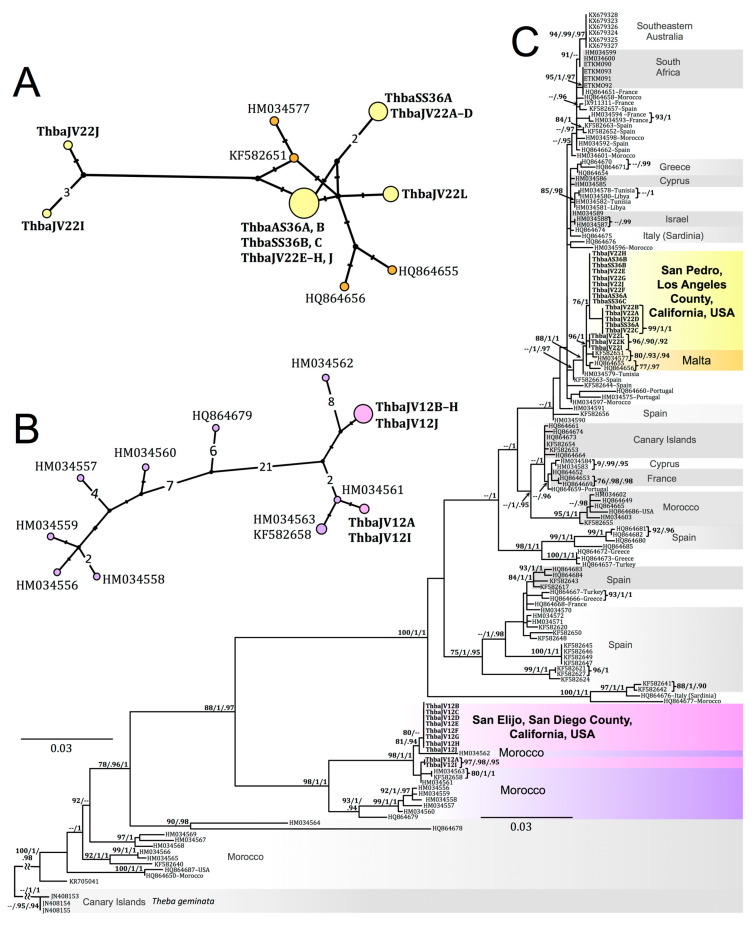
Haplotype networks and RAxML maximum likelihood phylogeny of *Theba pisana* mtDNA CO1 haplotypes (655 bp) from specimens collected in San Pedro, Los Angeles County and San Elijo Lagoon Ecological Reserve, San Diego County, California and available CO1 barcodes of *T. pisana* were native and introduced. (**A**) Median-joining network of 17 *T. pisana* CO1 haplotypes from specimens collected in San Pedro, Los Angeles County, California (in yellow) and 4 *T. pisana* CO1 haplotypes from Malta (in orange) [1,39,40]. (**B**) Median-joining network of 10 *T. pisana* CO1 haplotypes from specimens collected in San Elijo Lagoon Ecological Reserve, San Diego County, California (in pink) and 10 *T. pisana* CO1 haplotypes from Morocco (in purple) [1,39,40]. In both haplotype networks, circle size is proportional to the number of shared haplotypes in the dataset, and each hashmark represents a single nucleotide difference between haplotypes. (**C**) RAxML phylogeny based on partial CO1 sequence data (655 bp) from specimens collected in San Pedro, Los Angeles County and San Elijo Lagoon Ecological Reserve, San Diego County, with the outgroup *Theba geminata*, GenBank acc. no. JN408154, from Lanzarote, Canary Islands, Spain [39]. Color coding is consistent with haplotype networks. RA × ML bootstrap support values ≥75 are followed by Bayesian posterior probabilities ≥0.90 and Bayesian posterior probabilities for the dataset analyzed without the third codon position. Specimen names and locations in bold indicate *T. pisana* specimens collected in Southern California and sequenced for this study; GenBank accession numbers are in Table 1.

The most common CO1 haplotype from 17 San Pedro *T. pisana* specimens was found in 9 specimens collected near the south entrance of White Point Nature Preserve (WPNP) (BioSCAN 2019 and 2020 sites) and the White Point native plant garden; another haplotype was shared by specimens from the BioSCAN WPNP site and Marine Mammal Care Center site (Figure 8A). Two CO1 haplotypes were found among the 10 San Elijo-collected *T. pisana* specimens (Figure 8B).

Phylogenetic analyses of *T. pisana* CO1 haplotypes from San Pedro, San Elijo, and localities worldwide indicate *T. pisana* from sites in Los Angeles as most similar to specimens collected in Malta (e.g., GenBank acc. nos. HQ864655–56 [1], HM034577 [39], and KF582651 [40]) (Figure 8C and Supplementary Figure S1A), and San Elijo *T. pisana* as most similar to specimens collected in Morocco (e.g., GenBank acc. nos. HM034558–59, HM034561–63 [39], KF582658 [40]) (Figure 8C and Supplementary Figure S1B). Clades within this phylogeny (Figure 8C) also indicate affinities between *T. pisana* populations elsewhere in the world, e.g., South Africa and Southeast Australia, and the Canary Islands, France, Cyprus, and Morocco. Phylogenies have been uploaded to TreeBase at the following url: http://purl.org/phylo/treebase/phylows/study/TB2:S28531 (12 July 2021).

## 4. Discussion

### 4.1. Theba pisana in Southern California

Analysis of two molecular markers (CO1 and ITS2) revealed separate lineages of *T. pisana* in Southern California, indicating at least two introduction events that have resulted in present-day established populations in Los Angeles and San Diego Counties. Mitochondrial CO1 and 16S from *T. pisana* collected at several sites in San Pedro, Los Angeles County, show a single origin, the cause of which is unclear [22]. The spread of *T. pisana* to noncontiguous sites within San Pedro may have been facilitated unintentionally by humans and is likely to continue given the propensity of *T. pisana* to aestivate on structures, including vehicles. The similarity of San Pedro CO1 haplotypes to those from Malta could indeed indicate Maltese origin, but not all Mediterranean regions where *T. pisana* is prolific are represented in available data. CO1 haplotypes of *Theba pisana* from San Elijo, San Diego County are most similar to specimens from Morocco, and further sampling within San Diego County may reveal additional lineages with the same or different provenance. Morphological differences in jaws and mucous glands between San Pedro and San Elijo populations merit further investigation, as they may be useful in differentiating lineages or identifying cryptic taxa. 

These results contribute to a growing body of literature that documents repeated introduction of terrestrial gastropod taxa at sites around the world, e.g., *Ambigolimax valentianus* (Férussac, 1821) in Japan [68], *Arion subfuscus* (Draparnaud, 1805) in the U.S. [69,70], *Cepaea nemoralis* (Linnaeus) in North America [71,72], *Cornu aspersum* (Müller, 1774) in North and South America [73,74,75], *Deroceras invadens* Reise, Hutchinson, Schunack and Schlitt, 2011 in Europe and North America [76], *Helix* spp. in Europe [77], and *Lissachatina fulica* (Bowdich, 1822) in South America [78,79]. Such studies also highlight the phenomenon of biotic homogenization [80], identify anthropogenic pathways of introduction [81], may improve protocols for species interception and quarantine, and inform strategies for invasive species eradication or control [81]. Likewise, the distinct parentage of *T. pisana* in Southern California could be an important component of studies focused on diversification in the genus *Theba* [82] and varied investigations of genetic drift [83] and the founder effect [84], resource use [85], niche partitioning [86] and adaptation [87,88,89] in introduced gastropods. We anticipate that the CO1 barcodes and 16S and ITS2 sequences from *T. pisana* specimens generated for this study will be useful in assessing the provenance of new populations and interceptions of *T. pisana* in California, elsewhere in North America, and worldwide. 

The K2P genetic distance between the CO1 barcode fragments of *T. pisana* from San Pedro and San Elijo is 13%, which, in many other taxa, would be enough to designate them as separate species [90,91,92]. However, in the stylommatophora (land snails and slugs), intraspecific CO1 genetic distance can be as high as 30% [93], while interspecific genetic distance may be 1–3% [94,95]. Although a taxonomic treatment of *T. pisana*, its subspecies, and/or the identification of cryptic taxa are beyond the scope of this study, the preliminary integrative taxonomic work herein may provide a basis on which diversity within *T. pisana* and its lineages are further investigated [39].

### 4.2. Non-Californian Theba pisana

For *T. pisana* outside of California, analysis of CO1 barcode haplotypes indicates putative source populations in locations where it is introduced. For example, a monophyletic Southeastern Australian clade was sister to a polytomy of several South African haplotypes indicating a possible South African origin for SE Australian *T. pisana*, a scenario discussed by Däumer et al. [1]. The presence of France, Morocco, Spain, and the Canary Islands throughout the phylogeny highlights the remarkable diversification of *T. pisana* within its range and the complex history of its colonization and re-introduction at these localities [1]. Undoubtedly, and as discussed elsewhere [1,39], greater geographic sampling would facilitate determining source populations of *T. pisana* at other sites where it is introduced. This is particularly relevant for localities with *T. pisana* populations not represented in this dataset, for example, Argentina [2].

### 4.3. Mucous Glands

Mucous gland and jaw morphology were different between sites, with San Elijo (San Diego County) specimens having bi-lobed mucous glands and pronounced jaw ridges, and San Pedro (Los Angeles County) specimens having single-lobed mucous glands and less pronounced jaw ridges. According to several authors, mucous gland size (e.g., small vs. large), thickness (e.g., slender vs. thick) but not lobe morphology (e.g., single vs. bi-lobed) is variable within *T. pisana* [3,44]. Published drawings of the reproductive system in *T. pisana* and related lineages and varieties (e.g., *T. pisana* from Israel, *T. andalusica* from Spain, *T. p. almogravensis* from Portugal) depict only cylindrical, single-lobed mucous glands [3,42,44,96]. Likewise, Hesse [66] illustrates mucous glands as only single-lobed in specimens from Egypt, Lebanon, and Spain, but references bi-lobed mucous glands in *T. pisana* having been found by the malacologist, Moquin-Tandon, (in German: “Nach Moquin-Tandon sollen die glandulae mucosae zuweilen gespalten sein; mir ist ein solcher Fall nicht vorgekommen”). However, in Moquin-Tandon’s treatment of *T. pisana* (as *Helix pisana*), only single-lobed mucous glands are illustrated (in his Plate 19, Figure 16) [97].

Confounding efforts to distinguish if mucous gland morphology is lineage or species-specific is that reproductive morphology in *T. pisana* is not well documented throughout ontogeny [3]. Although some authors have indicated that “local forms” of *T. pisana* may be distinguished based on genitalia [3,96], ontogenetic series of their morphology is lacking. Without an assessment of the reproductive system in *T. pisana* throughout its maturation, it is unknown if mucous glands develop from bi-lobed to single-lobed or vice versa, though we suspect they do not. In samples analyzed herein, *T. pisana* collected in June from the White Point Nature Preserve have mostly immature genitalia with single-lobed mucous glands, which remained single lobed by October but had grown in size. Additionally and importantly, what we interpret as intermediate reproductive maturity in San Elijo specimens may actually be mature for that lineage. Therefore, although not explicitly tested, mucous gland morphology may be a lineage-specific trait in *T. pisana*.

### 4.4. Jaw

The morphology of the single jaw in *T. pisana* differed between specimens from San Pedro and San Elijo sites in the prominence of ridges, measured as the ratio of jaw ridge length to jaw length. It is not clear if this difference is lineage-specific and taxonomically valuable or an artifact of ontogeny. Both Moquin-Tandon [97] and Hesse [66] illustrate jaw morphology in *T. pisana*: Moquin-Tandon presents juveniles as having jaws with two ridges, while adults have three (his Plate 19, Figures 9 and 10). Hesse [66] characterized the jaw as having 2–4 ridges, and of 56 specimens he examined, 66% had two ridges, and most of the remaining had three. Hesse also found that in a sample of 40 putative juveniles with shell sizes from 6–14 mm: 26 jaws had two ridges, 13 had three ridges, and one had four ridges [66]. Despite the detail of Hesse’s observations, it is unclear if variation in jaw ridge number is a consequence of ontogeny or varies among same-aged *T. pisana* individuals within a lineage. Notably, Moran [42] found that a proportion of *T. pisana* within populations in Israel retained immature genitalia despite being of adult age; it is unknown if jaw ridge number varied in these developmentally arrested snails. In our small sample, the largest jaws from the most developmentally mature *T. pisana* collected in San Pedro (from October 2020, Figure 6D,I) had two prominent jaw ridges, which contradicts Moquin-Tandon [97] and may indicate that jaw ridge number is unrelated to ontogeny. Additionally, our finding that jaw ridges (regardless of number) were more prominent and in greater relief in San Elijo *T. pisana* than in those from San Pedro may indicate a lineage-specific trait that merits further investigation.

### 4.5. Shell and Radula

Highly variable banding patterns and shell pigmentation in *T. pisana* were observed herein but not analyzed between or within sites. For a detailed treatment and discussion of shell banding, see Cain [98], Cowie [99], and Köhler et al. [89]. Radular morphology was consistent with illustrations and descriptions by other authors [66,67] and was not significantly different between *T. pisana* from Los Angeles and San Diego sites.

### 4.6. Pathway(s) of Introduction into California

The means by which *T. pisana* was introduced to Southern California are uncertain, though many pathways are possible. *Theba pisana* has been regularly intercepted in imports since the 1930s at ports in the U.S., in cargo destined for California, and in goods coming through Southern California ports (e.g., Los Angeles, Long Beach, San Diego). From the 1930s–1980s, imported goods documented to contain *T. pisana* individuals include hyacinth bulbs, lilies and other flowers, endive, broad bean, rosemary, sand, soil, hides, tile, wood, and straw [100,101,102,103,104,105]. *Theba pisana* has also been recovered living in military cargo, automobiles, and baggage mostly from circum-Mediterranean countries [100]. Two *T. pisana* individuals intercepted separately in imported goods in Minnesota, USA, and included in the phylogeny by Däumer et al. [1] (GenBank acc. nos. HQ864686 and HQ864687) appear to both be of Moroccan origin, though not closely related. Therefore, a Maltese and/or Moroccan lineage of *T. pisana* in Southern California could have been due to snails from a variety of imports, making its introduction potentially human-mediated and inadvertent. 

However, local narratives in Southern California posit that *T. pisana* was first introduced intentionally to La Jolla, San Diego County from Italy, or specifically, Sicily [12,13]. The importer, as the story goes, was a La Jolla resident and/or Italian immigrant who intended to raise the snails for eating and/or selling, but the snails escaped or were discarded and then proliferated [20,22]. Indeed, *T.* *pisana* is common in Italy [106] and is eaten especially in Sicily [107], where it is prepared as ‘Lumache siciliane’ (Italian for ‘snails Sicilian’) or ‘Babbaluci alla palermitana’ (Sicilian for ‘snails Palermo style’) and is particularly popular during the summer feast of Santa Rosalia [108,109]. Because we did not include La Jolla-collected specimens in this study, we cannot directly test the plausibility of this narrative, assuming that *T. pisana* from La Jolla in the 1920s survived attempts at eradication. Additionally and importantly, our dataset does not include specimens from Sicily or mainland Italy, making the ability to test this Italian-origin hypothesis untenable even if we had *T. pisana* from La Jolla. The only Italian CO1 haplotypes in our dataset are from the island of Sardinia [1] (GenBank acc. nos. HQ864674–76) and these do not cluster with San Pedro or San Elijo CO1 haplotypes. Therefore, although San Elijo *T. pisana*, which is the only San Diego County-collected population in our dataset, has Moroccan parentage based on our analyses, we cannot reject a potential Italian origin for *T. pisana* elsewhere in San Diego County.

Regarding the Moroccan affinity of San Elijo *T. pisana*: it is possible that the present-day population we sampled descended from snails brought to northern San Diego County from Morocco for culinary purposes, as *T. pisana* is sometimes used in Moroccan preparations of Marrakech snail soup, known in Arabic as ‘babbouche’ [110]. However, this may not be a particularly likely scenario because babbouche is traditionally prepared with land snails in the genus *Otala*. It is also possible that *T. pisana* was intentionally introduced from elsewhere in the Mediterranean where it is eaten, e.g., Andalusia, Spain, where it is prepared as “caracoles chicos en caldo” (Spanish for ‘small snails in broth’), and that *T. pisana* from Spain and Morocco share haplotypes not present in our dataset. Speculations notwithstanding, the human-mediated pathway of introduction for San Elijo *T. pisana* remains unresolved.

All three molecular loci (mitochondrial CO1, 16S, and nuclear ITS2) analyzed from San Pedro, Los Angeles-collected *T. pisana* indicate a single common origin. These San Pedro sequences were most closely related to each other and were most similar to specimens collected on the Mediterranean island of Malta in the CO1 haplotype. We do not know if previously established populations of *T. pisana* in San Pedro, e.g., the 1935 populations that were supposedly eradicated [29], had a different parental origin or if some individuals survived and are the ancestors of the populations sampled in 2019 and 2020 for this study. Unfortunately, the malacology collections of NHMLAC do not include specimens from these 1935 populations or the Manhattan Beach population of the 1960s. While whole snail specimens collected from the 1930s–1960s could have been preserved in formalin, making DNA extraction potentially difficult, the morphology of jaws and reproductive parts would have been well preserved and could have been compared to living populations. Therefore, we reiterate recent appeals from several authors [111,112] to collect introduced species from urban and developed environments and make them available for study in museum collections.

In San Pedro, the maritime community of the early 20th century comprised a sizable Italian immigrant population from the islands of Ischia and Sicily [113,114]. Today, this population constitutes the largest Italian-American enclave in Los Angeles County [115]. It is possible that the *T. pisana* populations that proliferated in southeastern San Pedro in the 1930s were a consequence of Italian immigrants bringing this snail to the region as an edible delicacy, similar to the origin story of La Jolla *T. pisana* in the 1920s. In our dataset, the closest locality to Sicily from which we have CO1 haplotypes is Malta. Without Sicilian *T. pisana* haplotypes for comparison, we do not know if Maltese and Sicilian *T. pisana* share haplotype(s) that could indicate a possible Sicilian origin for present-day San Pedro snails that were not eradicated in the 1930s. Of course, and in addition to other alternative scenarios, *T. pisana* could have been introduced intentionally or accidentally to San Pedro from Malta after the eradication of the 1930s *T. pisana* infestation.

## 5. Conclusions

The establishment of two genetically divergent *T. pisana* lineages in Southern California indicates this species’ diversity and success were nonnative. Although its pathways of introduction into Los Angeles and San Diego Counties are not clear, the provenance of these lineages may be elucidated by greater sampling of *T. pisana* throughout Italy and in other regions where it is common but not represented in our dataset. Likewise, greater sampling throughout Southern California, especially within San Diego County, may reveal additional introduction events. The observation that the jaw and mucous gland morphology in *T. pisana* were distinct between Los Angeles and San Diego populations could inform future systematic treatments of *T. pisana* and merits further investigation. With the knowledge that multiple *T. pisana* lineages have proliferated in Southern California, subsequent studies could assess associated phenomena, such as introgression, niche partitioning, adaptation, and could inform efforts for species control.

## Figures and Tables

**Table 1:** Data for *Theba pisana* (Müller, 1774) specimens collected from Los Angeles and San Diego counties, California, USA and used in this study.

LACM Lot Number	Collection Date	Isolate Name	GenBank Accession Number			Reproductive System	Collection Locality	
			CO1	ITS2	16S			
182358	1-Jun-2019	ThbaS36A	MW831635	MW832220	MW847212	100% Immature (*n* = 5)	San Pedro, Los Angeles County, California	White Point Nature Preserve, BioSCAN site, south entrance 33.716, −18.316
		ThbaSS36B	MW831647	MW832226	MW847224			
		ThbaSS36C	MW831648	MW832227	MW847225			
		ThbaAS36A	MW831640	MW832224	MW847217			
		ThbaAS36B	MW831641	MW832225	MW847218			
182330	22-Jun-2020	ThbaJV22A	MW831636	MW832221	MW847213	100% Immature (*n* = 15)		Marine Mammal Care Center, 33.712, −118.295
		ThbaJV22B	MW831637	MW832222	MW847214			
		ThbaJV22C	MW831638	MW832223	MW847215			
		ThbaJV22D	MW831639	–	MW847216			
182329	22-Jun-2020	ThbaJV22E	MW831642	–	MW847219	100% Immature (*n* = 15)		White Point Nature Preserve, south entrance, 33.716, −118.316
		ThbaJV22F	MW831643	–	MW847220			
		ThbaJV22G	MW831644	–	MW847221			
		ThbaJV22H	MW831645	–	MW847222			
182331	22-Jun-2020	ThbaJV22I	MW831651	–	MW847228	100% Immature (*n* = 15)		White Point Nature Preserve, native plant garden, 33.716, −118.315
		ThbaJV22J	MW831646	–	MW847223			
		ThbaJV22K	MW831650	–	MW847227			
		ThbaJV22L	MW831649	–	MW847226			
182342	17-Oct-2020	–	–	–	–	7% Immature 93% Mature (*n* = 15)		White Point Nature Preserve, grassland loop trail, 33.715, −118.311
182335	12-Sept-2020	ThbaJV12A	MW831652	MW832228	–	20% Immature 80% Intermediate? (*n* = 15)	Solano Beach/Encinitas, San Diego County, California	San Elijo Lagoon Ecological Reserve, Santa Carina trail, 33.0077, −117.2596
		ThbaJV12B	MW831654	MW832230	–			
		ThbaJV12C	MW831655	MW832231	–			
		ThbaJV12D	MW831656	MW832232	–			
		ThbaJV12E	MW831657	MW832233	–			
		ThbaJV12F	MW831658	MW832234	–			
		ThbaJV12G	MW831659	–	–			
		ThbaJV12H	MW831660	MW832235	–			
		ThbaJV12I	MW831653	MW832229	–			
		ThbaJV12J	MW831661	–	–			

**Table 2:** Estimates of K2P genetic distance in *Theba pisana* CO1 mtDNA within and between sites in California (San Pedro, Los Angeles County and San Elijo Lagoon Ecological Reserve, San Diego County) and elsewhere in the world (see Figure 8C).

	San Pedro *T. pisana*	San Elijo *T. pisana*	All *T. pisana*
San Pedro *T. pisana* (*n* = 17)	0.004		
San Elijo *T. pisana* (*n* = 10)	0.131	0.004	
all other *T. pisana* (*n* = 128)	0.059	0.127	0.087

## Data Availability

Phylogenies may be found at TreeBase: http://purl.org/phylo/treebase/phylows/study/TB2:S28531 (accessed on 13 April 2021).

## Supplementary Materials

The following are available online at https://www.mdpi.com/article/10.3390/insects12080662/s1, Figure S1: Close-up of Figure 8.
