# Supplementary figures and images for: Multiple Introductions of the Pestiferous Land Snail Theba pisana (Müller, 1774) (Gastropoda: Helicidae) in Southern California
Source: Insects. 2021 Jul 21;12(8):662. doi: 10.3390/insects12080662 (PMC8396441; doi:10.3390/insects12080662)

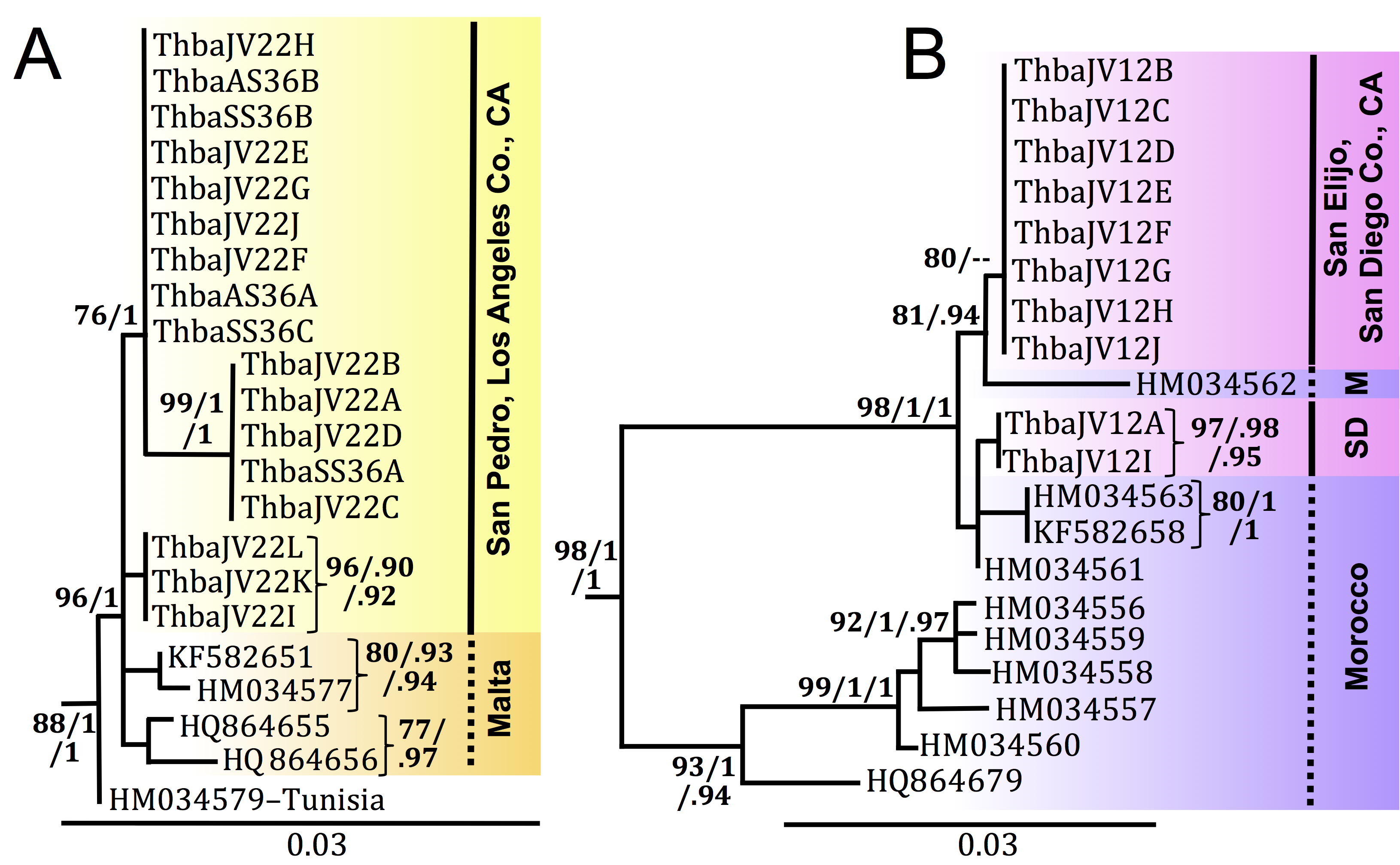

Supplement: Supplementary file 1 [file insects-12-00662-s001.zip › insects-1297688-supplementary.png]
